# A Review: Halogenated Compounds from Marine Actinomycetes

**DOI:** 10.3390/molecules26092754

**Published:** 2021-05-07

**Authors:** Cong Wang, Weisheng Du, Huanyun Lu, Jianzhou Lan, Kailin Liang, Shugeng Cao

**Affiliations:** 1 Key Laboratory of Chemistry and Engineering of Forest Products, State Ethnic Affairs Commission, Guangxi Key Laboratory of Chemistry and Engineering of Forest Products, Guangxi Collaborative Innovation Center for Chemistry and Engineering of Forest Products, Guangxi University for Nationalities, Nanning 530006, China; duweisheng2021@163.com (W.D.); luhuanyun2020@163.com (H.L.); lanjianzhou1576@163.com (J.L.); liangkailin1@163.com (K.L.); 2 Department of Pharmaceutical Sciences, Daniel K. Inouye College of Pharmacy, University of Hawai’i at Hilo, Hilo, HI 96720, USA

**Keywords:** marine actinomycetes, natural products, chemical structures, halogenated compounds

## Abstract

Marine actinomycetes, *Streptomyces* species, produce a variety of halogenated compounds with diverse structures and a range of biological activities owing to their unique metabolic pathways. These halogenated compounds could be classified as polyketides, alkaloids (nitrogen-containing compounds) and terpenoids. Halogenated compounds from marine actinomycetes possess important biological properties such as antibacterial and anticancer activities. This review reports the sources, chemical structures and biological activities of 127 new halogenated compounds originated mainly from *Streptomyces* reported from 1992 to 2020.

## 1. Introduction

Marine actinomycetes are a rich source of biologically active compounds, which have been widely studied worldwide. They can efficiently produce different secondary metabolites including simple benzene derivatives, polyketides and complex cyclic peptides. These secondary metabolites exhibit a wide range of biological activities including antibacterial, antifungal, anticancer and enzyme inhibition. Most of marine actinomyces were *Streptomyces* species, but rarer actinomycetes genera have been reported in the past twenty years. Consequently, more novel natural products including new halogenated compounds have been isolated in recent years. According to a review on marine microbial natural products from 2010 to 2013 [1], secondary metabolites from marine actinomycetes possess various structures, including terpenes, peptides, polyketides, alkaloids and halogenated molecules [2]. Due to the high concentration of chloride and bromine ions in seawater, marine actinomycetes usually produce more halogenated compounds than those of their terrestrial counterparts. The majority of the marine halogenated compounds showed certain kind of biological properties including antibacterial and anticancer activities [3]. This review focuses on the sources of marine actinomycetes, structures and biological activities of 127 new halogenated compounds derived from marine-derived actinomycetes from 1992 to 2020.

## 2. Halogenated Compounds from *Streptomyces* Species

### 2.1. Sponges-Associated Streptomyces sp.

**Figure 1:**
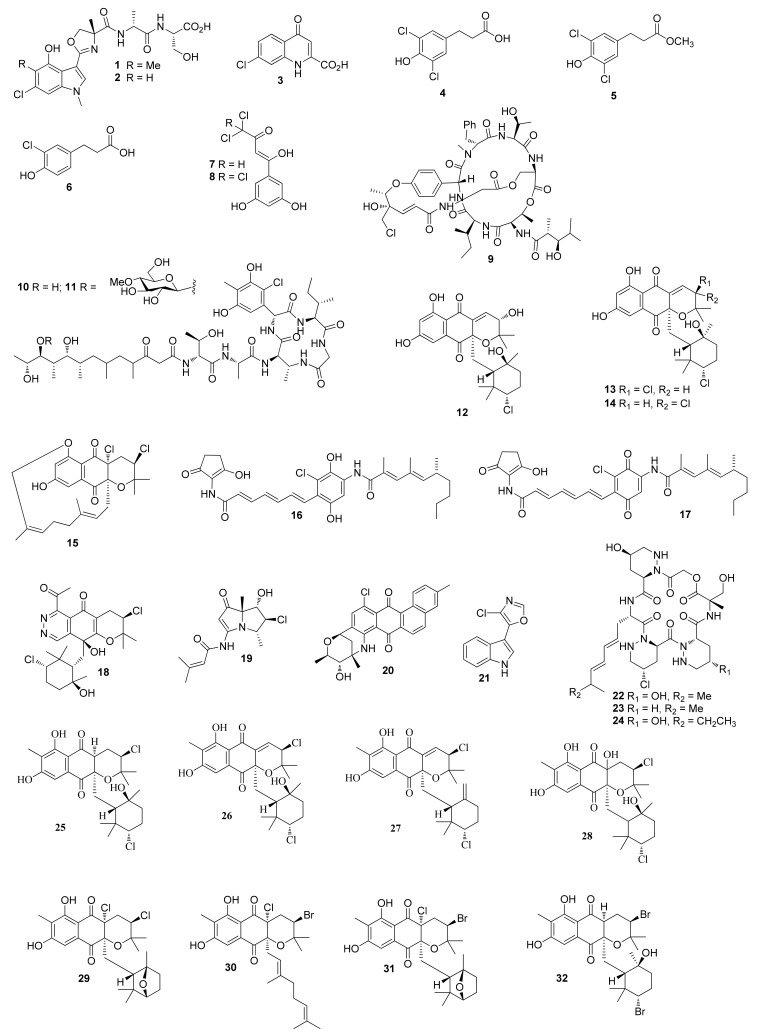
Structures of compounds **1**–**32**.

Two indole-containing peptides JBIR-34 (**1**) (Figure 1) and JBIR-35 (**2**) were obtained from *Streptomyces* sp. Sp080513GE-23 strain collected from a marine sponge, *Haliclona* sp. [4]. The nonribosomal peptidesan has an unusual 4-methyloxazoline moiety. Experiments showed that the methyl group comes from alanine rather than methionine [5]. Ageloline A (**3**), a new chlorinated quinolone separated from a fermentation of *Streptomyces* sp. SBT345, showed antioxidant effect and reduced oxidative stress and genomic damage induced by an oxidative stress inducer 4-nitroquinoline-1-oxide [6]. Compound **3** also inhibited the growth of *Chlamydia trachomatis* with an IC_50_ value of 9.54 μM [7]. New 3-phenylpropanoic acids 3-(3,5-dichloro-4-hydroxyphenyl) propanoic acid (**4**), 3-(3,5-dichloro-4-hydroxyphenyl) propanoic acid methyl ester (**5**) and 3-(3-chloro-4-hydroxyphenyl) propanoic acid (**6**) were isolated from *Streptomyces coelicolor* LY001, which demonstrated a broad spectrum of antibacterial activities with MIC values ranging from 16 to 250 μg/mL [8].

### 2.2. Corals-Associated Streptomyces sp.

Strepchloritides A (**7**) and B (**8**) were separated from a culture of *Streptomyces* sp. OUCMDZ-1703 and were cytotoxic against MCF-7 with IC_50_ values of 9.9 and 20.2 μM, respectively [9]. 

### 2.3. Streptomyces sp. from Other Marine Animals

A new depsipeptide salinamide B (**9**) was isolated from *Streptomyces hygroscopicus*. Compound **9** exhibited inhibitory activity against *Streptococcus pneumoniae* and *Staphylococcus pyrogenes*, with MIC values of 4 and 2 μg/mL, respectively. Compound **9** also exhibited 83% inhibition of edema with a testing dose of 50 pg/ear [10]. Polyketide–cyclic peptide hybrid metabolites totopotensamides A (**10**) and B (**11**) were separated from *Streptomyces* sp. 1053U.I.1a.1b [11]. New napyradiomycin, MDN-0170 (**12**) was purified from *Streptomyces* sp. strain CA-271078 [12]. *Streptomyces sp.* strain CA-271078 yielded napyradiomycin analogs napyradiomycin B7a, napyradiomycin B7b and napyradiomycin D1 (**13**–**15**), which were cytotoxic against HepG-2 tumor cell line with IC_50_ values of 41.7, 109.5 and 14.9 μM, respectively. Compounds **13** and **15** showed anti-bacterial activity against methicillin resistant *Staphylococcus aureus* (MRSA) and *Mycobacterium tuberculosis* with MIC values in the range of 12 to 48 μg/mL [13].

### 2.4. Streptomyces sp. from Marine Sediments

Cultivation of *Streptomyces* sp. M045 afforded chinikomycins A-B (**16**–**17**). Compound 1**6** inhibited tumor cell lines MAXF 401NL, MEXF 462NL and RXF 944L with IC_50_ values of 2.41, 4.15 and 4.02 µg/mL, respectively. Compound **17** inhibited MAXF 401NL with an IC_50_ value of 3.04 µg/mL [14]. An unusual meroterpenoid phthalazinone azamerone (**18**) was isolated from *Streptomyces* sp. CNQ766 [15]. In the synthesis of azamerone, Lewis-acid-induced cyclization, enantioselective synthesis of an epoxysilane and the formation of the pyridazine ring were three key steps [16]. A bohemamine-type pyrrolizidine alkaloid 5-chlorobohemamine C (**19**) was obtained from a culture of *Streptomyces* sp. CNQ-583 [17]. A pentacyclic C-glycoside marmycin B (**20**) was obtained from *Streptomyces* sp. CNH990, which displayed inhibitory activity against HCT-116 with an IC_50_ value of 1.09 μM [18]. *Streptomyces* sp. 04DH110 produced a new 3-substituted indole streptochlorin (**21**), which displayed cytotoxicity against human leukemia cells with an IC_50_ value of 1.05 μg/mL [19]. Total synthesis of streptochlorin started from indole, the synthetic product exhibited potential antifungal activity [20]. Three new hexadepsipeptides piperazimycins A–C (**22**–**24**) were isolated from *Streptomyces* sp. CNQ-593, which showed cytotoxicity against HCT-116 with an equal IC_50_ value of 76 ng/mL [21]. The formation of dipeptide moiety and a macrocyclization by an SN2 reaction were the two key steps in the synthesis of piperazimycin A [22]. Three new chlorinated dihydroquinones (**25**–**27**) were separated from culture of *Actinomycete strain* CNQ-525, and compounds **25**–**27** were active against MRSA and vancomycin-resistant *Enterococcus faecium* with MIC values of 1.95 and 3.90, 15.6 and 15.6 and 1.95 and 1.95 µg/mL, respectively. Compounds **25** and **26** showed cytotoxicity against HCT-116 with the IC_50_ values of 2.40 and 0.97 μg/mL, respectively [23].

**Figure 2:**
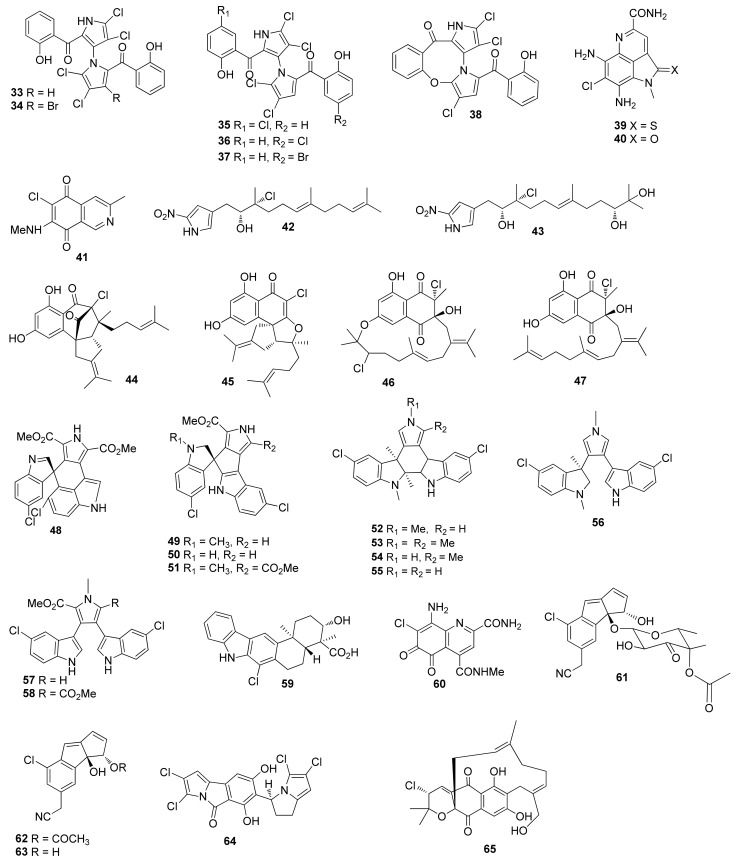
Structures of compounds **33**–**65**.

Heterologous expression of the CNQ-525-based nap biosynthetic cluster in *Streptomyces albus* produced 2-deschloro-2-hydroxy-A80915C (**28**), a new napyradiomycin [24]. Four napyradiomycin derivatives napyradiomycin CNQ525.510B (**29**), napyradiomycin CNQ525.538 (**30**), napyradiomycin CNQ525.554 (**31**) and napyradiomycin CNQ525.600 (**32**), were purified from the same strain, of which compounds **29**, **30** and **32** displayed inhibitory activity against HCT-116 with IC_50_ values of 17, 6 and 49 μM, respectively [25]. New marinopyrroles dimeric marinopyrroles A (**33**) (Figure 2) and B (**34**) were obtained from *Streptomyces* sp. CNQ-418 and were cytotoxic toward HCT-116 with IC_50_ values of 8.8 and 9.0 μM, respectively. Compounds **33** and **34** were also active against methicillin-resistant *S. aureus* (MRSA) with MIC values of 0.61 and 1.1 μM, respectively [26]. Marinopyrroles C–F (**35**–**38**) were obtained from the same strain, which displayed inhibitory activity against HCT-116 with IC_50_ values ranging from 1 to 5 μg/mL. Compound **35** was active against MRSA with an MIC value less than 1 μg/mL [27]. Ammosamides A (**39**) and B (**40**) were separated from *Streptomyces* sp. CNR-698, which showed cytotoxicity against HCT-116 cells with an equal IC_50_ value of 320 nM [28]. Ammosamides A and B were synthesized from 4-chloroisatin [29].

**Figure 3:**
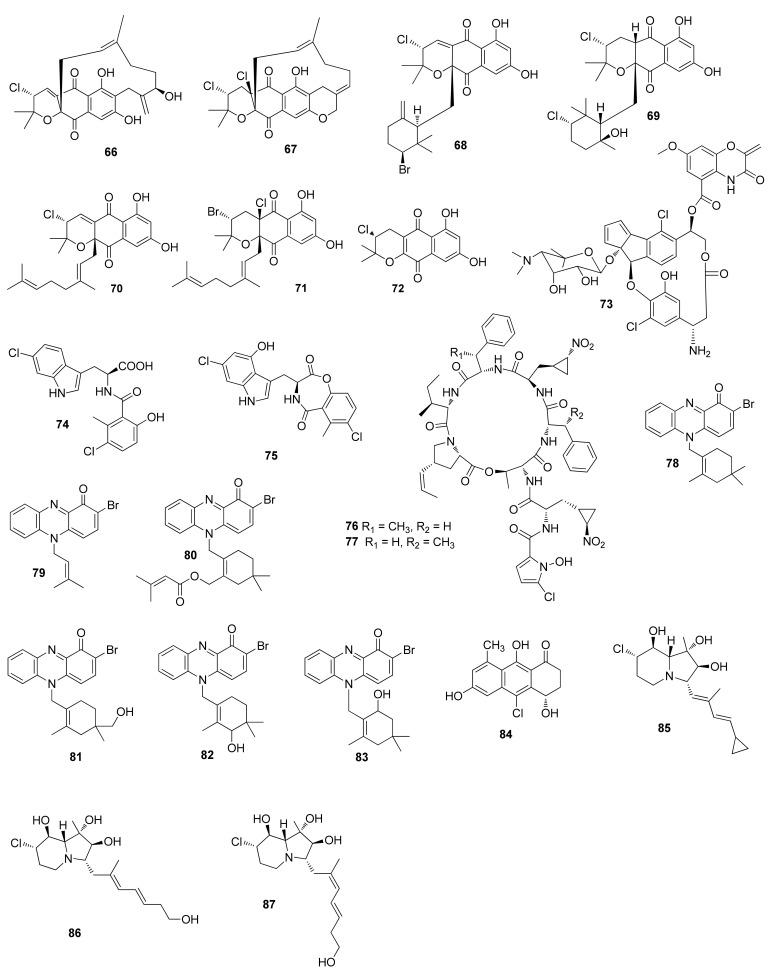
Structures of compounds **66**–**87**.

A cytotoxic compound, mansouramycin B (**41**) was isolated from the fermentation broth of *Streptomyces* sp. Mei37 [30]. Compound **41** was synthesized by using a new method through a catalytic acid-mediated cyclization of α-benzyl TosMIC derivatives [31]. *Streptomyces malaysiensis* CNQ-509 afforded nitropyrrolins C (**42**) and E (**43**), and **42** displayed cytotoxic activity against HCT-116 with an IC_50_ value of 31.0 μM [32]. *Streptomyces* sp. CNH-189 produced merochlorins A–D (**44**–**47**) [33]. Spiroindimicins A–D (**48**–**51**) [34], indimicins A–E (**52**–**56**), lynamicin F (**57**) and lynamicin G (**58**) were separated from *Streptomyces* sp. SCSIO 03032, among which **49**–**52** displayed cytotoxic activity against a panel of cancer cell lines with IC_50_ values ranging from 4 to 15 μM. Compound **53** also showed cytotoxicity toward MCF-7, with an IC_50_ value of 10.0 µM [35]. Merochlorins A and B were synthesized by heterologously produced enzymes and chemical synthesis [36]. (±)-Spiroindimicins B and C were synthesized, and central to the successful strategy was installing the spirocenter [37]. Chloroxiamycin (**59**), was isolated from *Streptomyces* sp. SCSIO 02999, which displayed antimicrobial activity against *E. coli* ATCC 25922, *S. aureus* ATCC29 213 and *B. subtilis* SCSIO BS01 with MIC values of 4, 8 and 64 μg/mL, respectively [38]. *Streptomyces variabilis* SNA-020 afforded an oxidatively ring opened ammosamide analog ammosamide D (**60**), which displayed cytotoxic activity against the MIA PaCa-2 with an IC_50_ value of 3.2 μM [39]. Cultivation of *Streptomyces* sp. CNT-179 strain afforded cyanosporasides C–E (**61**–**63**) [40]. Chlorizidine A (**64**) was isolated from *Streptomyces* sp. CNH-287, which showed cytotoxic activity against the HCT-116 adenocarcinoma cell line with an IC_50_ value of 3.2–4.9 μM [41]. (±)-Chlorizidine A was synthesized by decarboxylative coupling and late-stage oxidation, Reformatsky reaction and Mitsunobu reactions [42]. *Streptomyces* sp. CNQ-329 yielded five new halogenated napyradiomycins A and C–E (**65**–**68**) (Figure 3), of which compounds **65**, **67** and **68** exhibited inhibitory activity towards HCT-116 with IC_50_ values of 4.2, 16.1 and 4.8 μg/mL, respectively. Compound **65** displayed antibacterial activity against MRSA with an MIC value of 16 µg/mL [43]. Napyradiomycin F (**69**) from *Streptomyces* sp. CNH-070 showed inhibitory activity against HCT-116, with an IC_50_ value of 9.42 μg/mL [43].

*Streptomyces* sp. SCSIO 10,428 afforded three new napyradiomycins 4a-dechloronapyradiomycin A1 (**70**), 3-dechloro-3-brominapyradiomycin A1 (**71**) and 3-chloro-6,8-dihydroxy-α-lapachone (**72**). Compound **70** demonstrated antibacterial activity against *Staphylococcus aureus* ATCC 29213, *Bacillus subtilis* SCSIO BS01 and *Bacillus thuringensis* SCSIO BT01 with MIC values of 4, 4 and 8 μg/mL; **71** exhibited antibacterial activity against *Staphylococcus aureus* ATCC 29213, *Bacillus subtilis* SCSIO BS01 and *Bacillus thuringensis* SCSIO BT01 with MIC values of 0.5, 1 and 1 μg/mL; and **72** showed antibacterial activity against *Bacillus subtilis* SCSIO BS01 and *Bacillus thuringensis* SCSIO BT01 with MIC values of 8 and 16 μg/mL, respectively. Compound **70** also displayed inhibitory activity against SF-268, MCF-7, NCI-H460 and HepG-2 with IC_50_ values of 22.8 ± 0.3, 20.6 ± 0.1, 22.4 ± 0.1 and 21.8 ± 0.5 μM, respectively; **71** showed inhibitory activity against SF-268, MCF-7, NCI-H460 and HepG-2 with IC_50_ values of 11.5 ± 1.2, 16.2 ± 0.7, 18.1 ± 0.3 and 17.1 ± 1.0 μM, respectively; and **72** exhibited inhibitory activity against SF-268, MCF-7, NCI-H460 and HepG-2 with IC_50_ values of 23.8 ± 2.2, 71.1 ± 0.4, 127.1 ± 0.9 and 59.4 ± 0.7 μM, respectively [44]. C-1027 chromophore-V (**73**) was obtained from a marine-derived *Streptomyces* sp. ART5, which showed inhibitory activity against *Candida albicans* isocitrate lyase with an IC_50_ value of 37.9 μM.

Compound **73** also inhibited MDA-MB231 and HCT-116 with IC_50_ values of 0.9 and 2.7 μM, respectively [45]. Chlorinated alkaloids inducamides A (**74**) and C (**75**) were separated from *Streptomyces* sp. SNC-109-M3, of which compound **75** showed cytotoxicity against NSCLC cell line HCC44 with an IC_50_ value of 10 μM [46]. Inducamide A (**74**) was synthesized from 6-hydroxy-3-chloro-2-methylbenzoic acid and *L*-6-chlorotryptophan [47]. Inducamide C (**75**) is unstable and easy to rearrange [48].

Hormaomycins B (**76**) and C (**77**) were obtained from a marine-derived *Streptomyces* sp. SNM55. Compounds **76** and **77** were active against *S. aureus* ATCC 25923, *B. subtilis* ATCC 6633, *K. rhizophila* NBRC 12708, *S. pyogenes* ATCC 19615, *S. enterica* ATCC 14,028 and *P. hauseri* NBRC 3851 with MIC values of 7/7, 14/56, 0.4/0.23, 14/8, 29/114 and 29/14 μM, respectively [49]. Two new phenazines marinocyanins A and B (**78** and **79**) were isolated from *Streptomyces* sp. CNS284, which inhibited the TNF-α-induced NF-κB activity with IC_50_ values of 4.1 and 24.2 μM and suppressed the PGE2 production with IC_50_ values of 7.15 and 0.89 μM, respectively [50]. Compound **78** inhibited LPS-induced nitric oxide production with an IC_50_ value of 15.1 μM [50]. Compounds **78** and **79** showed cytotoxicity against HCT-116 cell with IC_50_ values of 0.049 and 0.029 μM and inhibited *S. aureus* and *C. albicans* with MIC values of 2.37/33.92 and 0.95/5.79 μg/mL, respectively [51]. Marinocyanins A and B were synthesized through the establishment of the N-substituted phenazin-1-one skeleton [52]. Four phenazinone named marinocyanins C–F (**80**–**83**) were purified from the marine actinomycete *Streptomycetaceae* CNS-284, which were active against *S. aureus* and *C. albicans* with MIC values ranging from 3.90 to 36.62 μg/mL. They also showed cytotoxicity against HCT-116 cell with IC_50_ values ranging from 0.078 to 17.14 μM [51]. A new tetrahydroanthracene alokicenone D (**84**) was isolated from the cultures of *Streptomyces* sp. HN-A101 [53]. New cyclizidine-type alkaloids cyclizidines D (**85**), H (**86**) and I (**87**) were purified from *Streptomyces* sp. HNA39. Compounds **86** and **87** exhibited inhibition against the ROCK2 protein kinase with IC_50_ values of 42 ± 3 and 39 ± 1 μM; **86** and **87** also showed cytotoxicity against PC-3 with IC_50_ values of 33 ± 1 and 17 ± 1 μM, respectively. Compound **87** demonstrated cytotoxicity against HCT-116 with an IC_50_ value of 40 ± 1 μM [54].

**Figure 4:**
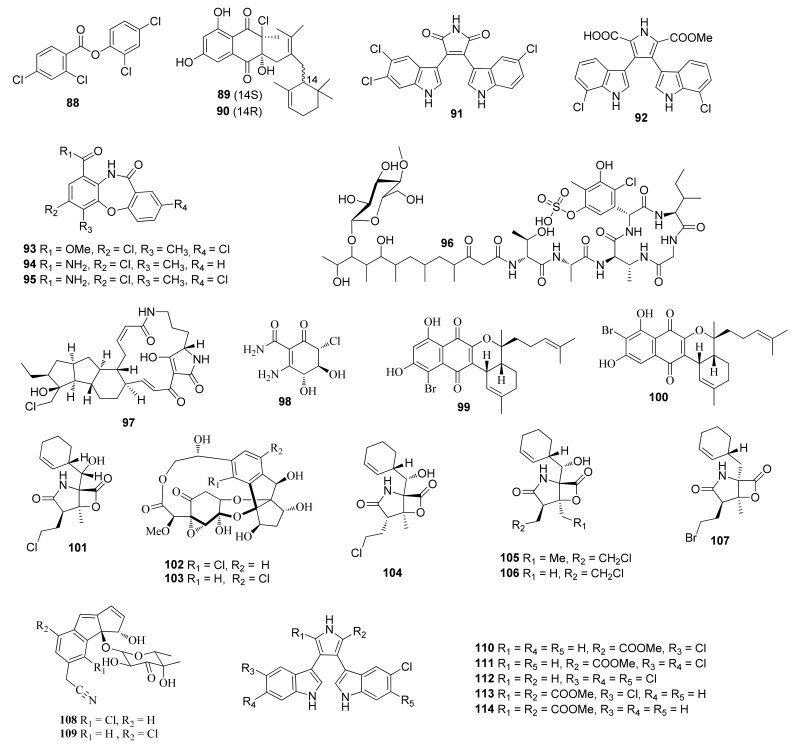
Structures of compounds **88**–**114**.

2,4-Dichlorophenyl 2,4-dichloro benzoate (**88**) (Figure 4) was obtained from *Streptomyces* sp. G212. Compound **88** was active against *C. albicans* with an MIC value of 64 μg/mL [55]. *Streptomyces* sp. CNH-189 afforded two new meroterpenoids merochlorins E (**89**) and F (**90**), which showed antibacterial activities against *S. aureus*, *B. subtilis* and *K. rhizophila* with MIC values ranging from 1 to 2 μg/mL [56]. Two new chlorinated bisindole alkaloids, dionemycin (**91**) and 6-OMe-7′,7″-dichorochromopyrrolic acid (**92**) were isolated from *Streptomyces* sp. SCSIO 11,791 [57]. Compound **91** displayed cytotoxic activity against MD1-MB-435, MDA-MB-231, NCI-H460, HCT-116, HepG2, and MCF10A with MIC values in the range of 3.1–11.2 μM. Compound **92** showed cytotoxic activity against human cancer cell lines MD1-MB-435, HCT-116, HepG2, and MCF10A with MIC values ranging from 2.9 to 19.4 μM.

### 2.5. Streptomyces sp. from Other Marine Sources

New dibenzoxazepinones mycemycins C–E (**93**–**95**) were separated from *Streptomyces olivaceus* FXJ8.012Δ1741 [58]. A sulfonate-containing analog totopotensamide C (**96**) was isolated from *Streptomyces pactum* SCSIO 02,999 [59]. One new polycyclictetramate macrolactam pactamide F (**97**) was also purified from *Streptomyces pactum* SCSIO 02,999 [60]. Cultivation of *Streptomyces* sp. ZZ502 afforded a new cyclohexene 3-amino-2-carboxamine-6(*R*)-chloro-4(*R*)-5(*S*)-dihydroxy-cyclohex-2-en-1-one (**98**) [61].

## 3. Halogenated Compounds from Other Marine Actinomycetes

### 3.1. Other Marine Sediments-Associated Actinomycetes

Actinomycete CNB-632 (sediment, the Tot-my Pines Estuary) yielded a sesquiterpenoid naphthoquinone marinone (99) that was active against *Bacillus subtilis* with an MIC value of 1 μg/mL [62]. An actinomycete strain (# CNH-099) produced isomarinone (**100**). Compound **100** displayed cytotoxicity against a colon carcinoma cell line HCT-116 with an MIC value of 8 μg/mL [63]. Salinosporamide A (**101**) with a γ-lactam-β-lactone bicyclic ring was isolated from *Salinospora* strain CNB-392 (later assigned as *Salinispora tropica*), which showed cytotoxicity against a panel of cancer cell lines with IC_50_ values less than 10 nM and exhibited prominent inhibition of the 20S proteasome [64]. Compound **101** entered phase I human clinical trials for the treatment of multiple myeloma three years after its discovery in 2003 [64]. Salinosporamide A was synthesized from (*R*)-pyroglutamic acid [65]. *Salinispora tropica* CNB-392 produced sporolides A (**102**) and B (**103**) [66]. Compound **103** exhibited inhibitory activity against HIV-1 reverse transcriptase [67]. Compound **103** was synthesized by ruthenium-catalyzed [2+2+2] cycloaddition and Diels–Alder-type reaction [68]. *Salinispora tropica* CNB-392 yielded salinosporamide F (**104**), salinosporamide I (**105**), salinosporamide J (**106**) and bromosalinosporamide (**107**). Compound **106** displayed RPMI 8226 and chymotrypsin-like activity with IC_50_ values of 52 and 250 nM, respectively [69]. Fermentation of *Salinispora pacifica* (designated CNS103) derived from sediments led to the identification of cyclopenta[a]indene glycosides cyanosporasides A and B (**108** and **109**). Compound **108** was cytotoxic against HCT-116 with an IC_50_ value of 30 µg/mL [70]. Lynamicins A–E (**110**–**114**) were afforded by *Marinispora* sp. NPS12745, which showed antibacterial activity against MRSA and vancomycin-resistant *E. faecium* with IC_50_ values in the range of 1.8–57.0 μg/mL [71].

**Figure 5:**
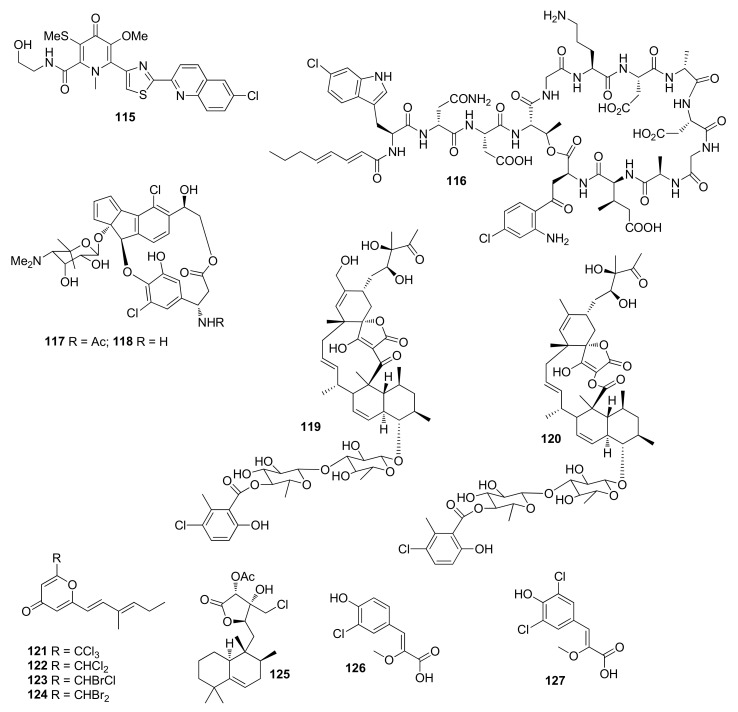
Structures of compounds **115**–**127**.

Lodopyridone (**115**) (Figure 5) from *Saccharomonospora* sp. (marine sediment, the La Jolla Submarine Canyon) showed cytotoxicity against HCT-116 cell line with an IC_50_ value of 3.6 μM [72]. *Saccharomonospora* sp. CNQ-490 afforded taromycin A (**116**), which exhibited antibacterial activity against MRSA and *Enterococcus faecalis* 613D with MIC values ranging from 6 to 100 μM [73]. Fijiolides A and B (**117** and **118**) were isolated from the cultures of bacterium of the genus *Nocardiopsis* CNS-653 (sediment sample, Fiji). Compound **117** showed QR1 activity and was active against TNF-R-induced NF-κB with an IC_50_ value of 0.57 μM. Compound **118** showed activity against TNF-R-induced NFκB [74].

Phocoenamicins B (**119**) and C (**120**) were isolated from *Micromonospora* sp. CA-214671, and both compounds showed a broad spectrum of antibacterial activities with MIC values ranging from 2 to 64 μg/mL [75].

### 3.2. Other Ascidian-Associated Actinomycetes

Halomadurones A–D (**121**–**124**) were obtained from *Actinomadura* sp. WMMB499 (ascidian *Ecteinascidia turbinata*), among which **123** and **124** showed activity against Nrf2-ARE [76]. A new halimane-type diterpenoid micromonohalimane B (**125**) was isolated from a culture of *Micromonospora* sp. WMMC-218, which inhibited MRSA with an MIC value of 40 μg/mL [77].

### 3.3. Other Marine Source-Associated Actinomycetes

Saccharochlorines A (**126**) and B (**127**) were isolated from *Saccharomonospora* sp. KCTC-19160, and both compounds showed BACE1 inhibition of 41.4 ± 3.6% and 32.0 ± 9.7%, respectively. at the same concentration of 50 μM (a positive control, isoliquiritigenin, 56.7% inhibition at 50 μM) [78].

## 4. Summary

**Figure 6:**
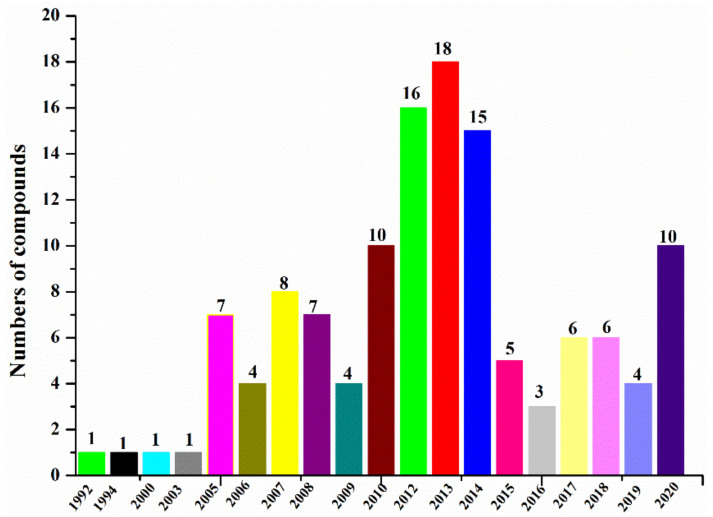
Numbers of new halogenated compounds from actinomycetes reported annually from 1992 to 2020.

According to the summary of halogenated compounds from marine-derived actinomycetes (Figure 6 and Table 1), the study of halogenated compounds from marine-derived actinomycetes could be traced back to 1992 when marinone (**99**) was purified from an actinomycete strain CNB-632 isolated from a sediment sample (Table 2) [62]. Since 2005, more new halogenated compounds from marine-derived actinomycetes have been isolated annually than ever before except for 2016. From 2010 to 2014 and in 2020, 10 or more new halogenated compounds were reported annually. By the end of 2020, 127 new halogenated compounds from marine-derived actinomycetes have been reported.

**Figure 7:**
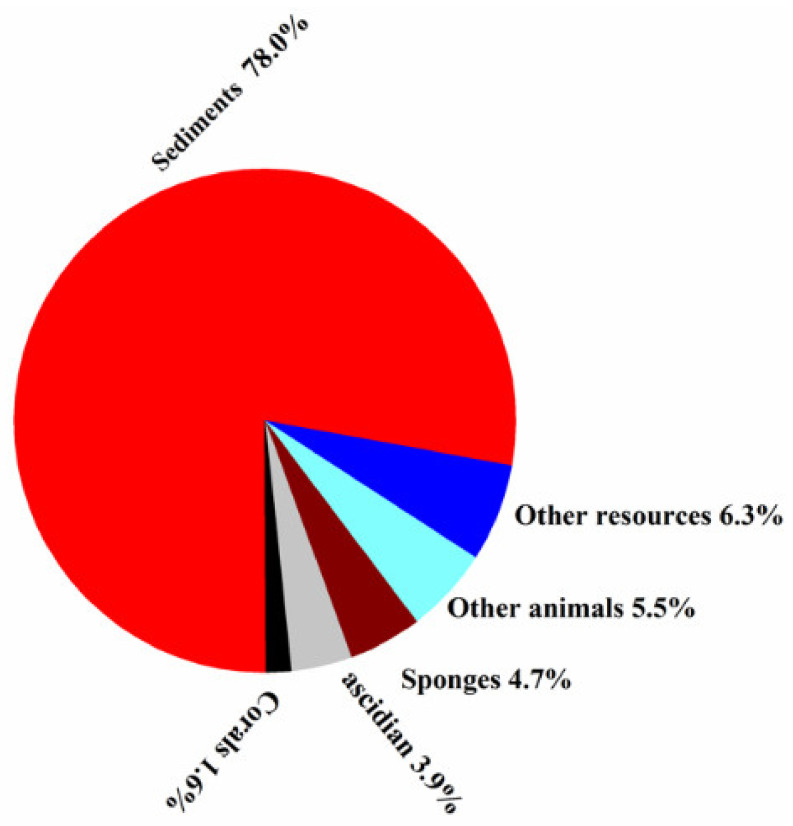
Percentages of new halogenated compounds from different sources of marine origins (1992–2020).

Sediments were the richest source of marine-derived actinomycetes, which produced about 78% of new halogenated compounds (Figure 7). It was reported that sediments are rich in nutrients, which can harbor an enormous quantity of microorganisms, including actinomycetes. It is worth mentioning that, the deeper and older the sediment is, the less abundant the microbes. Nevertheless, marine actinobacteria in sediments will keep providing opportunities for natural product research and natural product drug discovery.

**Figure 8:**
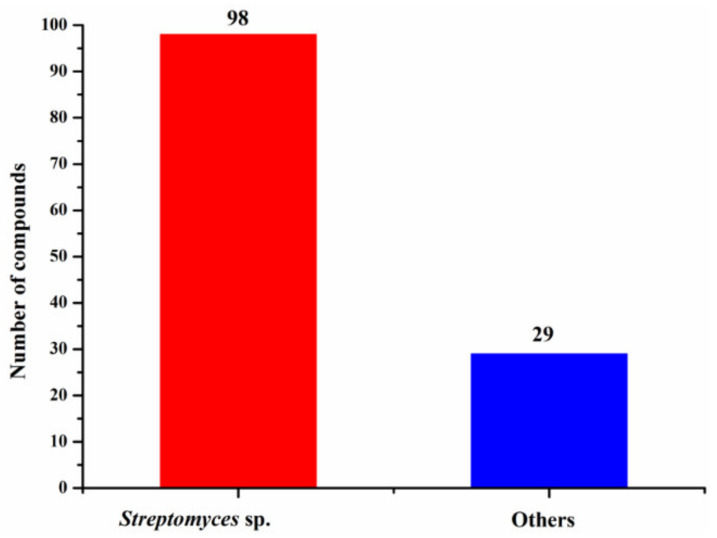
Numbers of new halogenated compounds from different marine actinomycetes (1994–2019).

**Figure 9:**
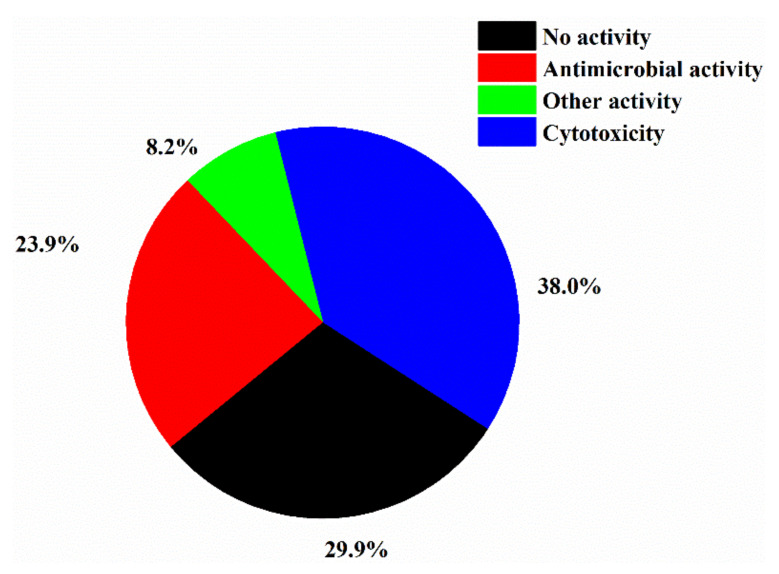
Activity of new halogenated compounds from marine actinomycetes (1992–2020).

Marine *Streptomyces* spp. had the highest occurrence of halogenated compounds (98/127 = 77%) (Figure 8), which might be due to their unique and diverse biosynthetic machinery, high halogenase activity or simply *Streptomyces* being the largest genus of Actinobacteria. Overall, 70.1% of halogenated compounds from marine actinomycetes is biologically active, and 37.3% and 24.6% of the halogenated compounds showed anticancer and antimicrobial activity, respectively (Figure 9).

**Figure 10:**
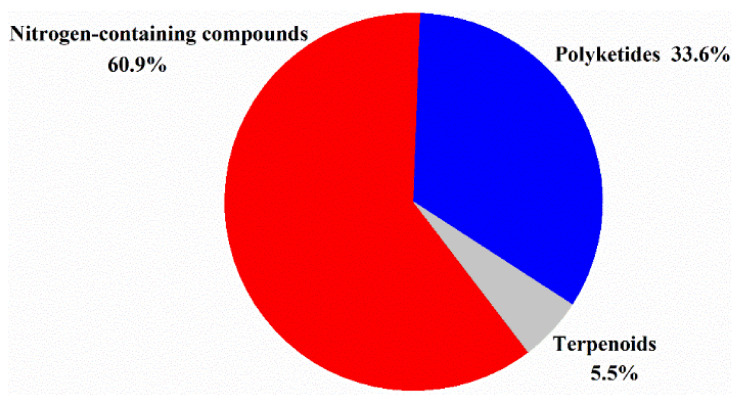
Structural classes of new halogenated compounds from actinomycetes (1992–2020).

**Figure 11:**
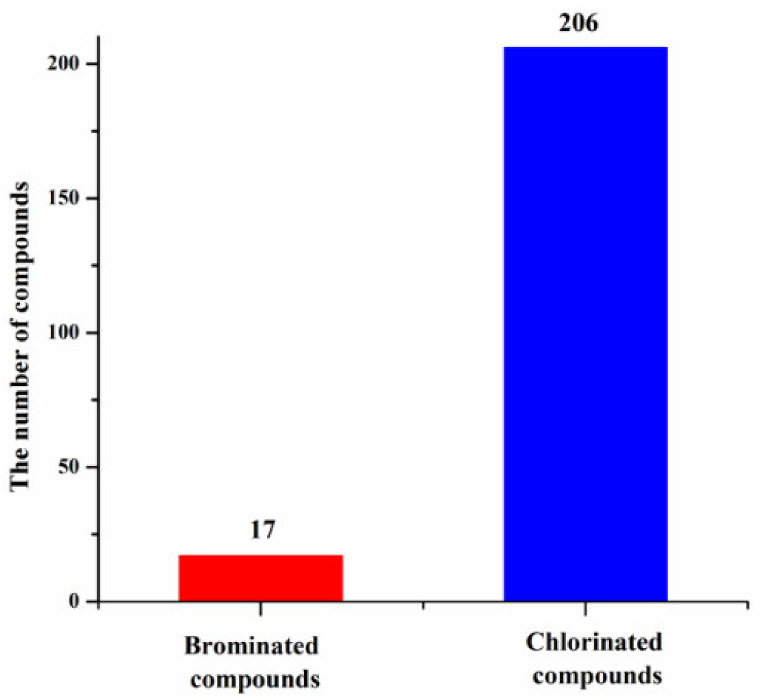
Proportion of new halogenated compounds from actinomycetes (1992–2020).

The structure types of the new halogenated compounds were diverse, which could be classified as nitrogen-containing compounds, polyketides and terpenoids. Nitrogen-containing compounds and polyketides were two main classes of compounds produced by marine actinomycetes (Figure 10). The number of chlorinated compounds generated by marine actinomycetes is 10 times more than that of brominated compounds (Figure 11), which may be related to the concentrations of chloride and bromide ions in the ocean. Fluorinated natural products were reported before, but no new fluorinated compounds were discovered from marine actinomycetes recently.

In short, marine actinomycetes have unique and diverse biogenetic machinery, which can produce different halogenated compounds with novel structure skeletons and various biological activities, and *Streptomyces* spp. from sediments are the main producers. Some halogen-containing drugs such as chloramphenicol, vancomycin, chlortetracycline, calicheamicin, rebeccamycin and complestatin have been developed from secondary metabolites isolated from terrestrial actinomycetes [3]. Marine natural products have higher success rate (1 in 3500) in drug discovery, compared with the industry average of 1 in 5000–10,000 compounds [79]. Therefore, halogenated compounds from marine actinomycetes are expected to be a promising source of lead compounds for natural product drug discovery.

## Figures and Tables

**Table 1:** The initial research on halogenated compounds from marine-derived actinomycetes.

First Producing Strain	Environment Source	Compound.	Time
*Streptomyces* sp. 1053U.I.1a.1b	*Lienardia totopotens*, Mactan Island, Cebu, Philippines	totopotensamides A (**10**) and B (**11**)	1994
Actinomycete CNB-632 (other marine actinomycetes)	Sediment sample, Tot-my Pines Estuary, La Jolla, CA	marinone (**99**)	1992

**Table 2:** Halogenated compounds isolated from marine-derived actinomycetes.

Compound	Producing Strain	Environment Source	Bioactivity	Ref.
**1–2**	*Streptomyces* sp. Sp080513GE-23	*Haliclona* sp. Sponge, Chiba, Japan	/	[4,5]
**3**	*Streptomyces* sp. SBT345	*Agelas oroides* sponge, Mediterranean Sea	Antioxidant and antichlamydial effects	[6,7]
**4–6**	*Streptomyces coelicolor* LY001	sponge *Callyspongia siphonella*, the Saudi Red Sea	Antibacterial activity	[8]
**7–8**	*Streptomyces* sp. OUCMDZ-1703	Unidentified soft coral, Weizhou Island, Guangxi, China	Cytotoxicity	[9]
**9**	*Streptomyces hygroscopicus*	Jellyfish *Cassiopeia xamachana*, Florida Keys	Antibacterial, Anti- inflammatory activity	[10]
**10–11**	*Streptomyces* sp. 1053U.I.1a.1b	*Lienardia totopotens*, Mactan Island, Cebu, Philippines	/	[11]
**12–15**	*Streptomyces* sp. Strain CA-271078	Ascidian, the sea shore in Baía Ana Chaves, Sao Tome	**13–15**: Cytotoxicity **13**, **15**: Antibacterial activity	[12,13]
**16–17**	*Streptomyces* sp. M045	Sediment, Jiaozhou Bay, China	Cytotoxicity	[14]
**18**	*Streptomyces* sp. CNQ766	Sediment, Island of Guam	/	[15,16]
**19**	*Streptomyces* sp. CNQ-583	Sediment, Island of Guam	/	[17]
**20**	*Streptomyces* sp. CNH990	Sediment, Cabo San Lucas, Mexico.	Cytotoxicity	[18]
**21**	*Streptomyces* sp. 04DH110	Sediments, Ayajin Bay, East Sea of Korea	Cytotoxicity	[19,20]
**22–24**	*Streptomyces* sp. CNQ-593	Sediment, Island of Guam	Cytotoxicity	[21,22]
**25–32**	*Streptomcyces* sp. CNQ525	Sediment, La Jolla, CA	**26–27**, **29–30**, **32**: Cytotoxicity **25–27**: Antibacterial activity	[23,24,25]
**33–38**	*Streptomyces* sp CNQ-418	Sediment, La Jolla, CA	Cytotoxicity	[26,27]
**39–40**	*Streptomyces* sp. CNR-698	Sediment, Bahamas Islands	Cytotoxicity	[28,29]
**41**	*Streptomyces* sp. Mei37	Sediment, Jade Bay, German	Cytotoxicity	[30,31]
**42–43**	*S*. *malaysiensis* CNQ-509	Sediment, California	**42**: Cytotoxicity	[32]
**44–47**	*Streptomyces* sp. CNH-189	Sediment, Oceanside, California	/	[33]
**48–58**	*Streptomyces* sp. SCSIO 03032	Sediment, Bay of Bengal	**49–51**, **53**: Cytotoxicity	[34,35,36,37]
**59**	*Streptomyces* sp. SCSIO 02999	Sediment, South China Sea	Antibacterial activity	[38]
**60**	*Streptomyces variabilis* SNA-020	Sediment, Bahamas	Cytotoxicity	[39]
**61–63**	*Streptomyces* sp. CNT-179	Sediment, Bahamas	/	[40]
**64**	*Streptomyces* sp. CNH-287	Sediment, San Diego, CA.	Cytotoxicity	[41,42]
**65–68**	*Streptomyces* sp. CNQ-329	Sediment, San Diego, CA.	**65**, **67–68**: Cytotoxicity **65**: Antibacterial	[43]
**69**	*Streptomyces* sp. CNH-070	Sediment, Encinitas, California	Cytotoxicity	[43]
**70–72**	*Streptomyces* sp. SCSIO 10428	Sediment, Beihai, Guangxi, China	Antibacterial activity	[44]
**73**	*Streptomyces* sp. ART5	Sediment, East Siberian, Arctic Ocean	Cytotoxicity	[45]
**74–75**	*Streptomyces* sp. SNC-109-M3	Sediment, Vava’u, Tonga	**74**: Cytotoxicity	[46,47,48]
**76–77**	*Streptomyces* sp. SNM55	Sediment, Buan, Korea	Antibacterial activity	[49]
**78–83**	*Streptomycetaceae* CNS-284	Marine sediments, the Solomon Islands and in Palau	**78–79**: TNF-α-induced NFκB activity and antibacterial activity; **80**–**83**: Antibacterial activity and cytotoxicity	[50,51,52]
**84**	*Streptomyces* sp. HN-A101	Mangrove soil, Hainan, China	/	[53]
**85–87**	*Streptomyces* sp. HNA39	Marine sediment, Hainan, China	**86–87**: Cytotoxicity	[54]
**88**	*Streptomyces* sp. G212	Marine sediment, Quang Binh-Vietnam	Antifungl activity	[55]
**89–90**	*Streptomyces* sp. CNH-189	Sediment, near Oceanside, California.	Antibacterial activity	[56]
**91–92**	*Streptomyces* sp. SCSIO 11791	Sediment, South China Sea	cytotoxicity	[57]
**93–95**	*Streptomyces olivaceus* FXJ8.012Δ1741	a gntR gene-disrupted deep-sea strain	/	[58]
**96–97**	*Streptomyces pactum* SCSIO 02999	Sediment, South China Sea	/	[59,60]
**98**	*Streptomyces* sp. ZZ502	Seaweed *Ulva conglobatea* (Family Ulvaceae).	/	[61]
**99**	Actinomycete CNB-632	Sediment sample, Tot-my Pines Estuary, La Jolla, CA	Antibacterial activity	[62]
**100**	Actinomycete (strain # CNH-099)	Sediment, Batiquitos Lagoon, North of San Diego, CA	Cytotoxicity	[63]
**101–107**	*Salinospora* strain CNB-392(later assigned as *Salinispora tropica*)	Sediment, Chub Cay, Bahamas	**101**, **106**: Cytotoxicity **103**: inhibitory activity against HIV-1 reverse transcriptase **106**: chymotrypsin-like activity	[64,65,66,67,68,69]
**108–109**	*Salinispora pacifica* (designated CNS103)	Sediment, Palau	**108**: Cytotoxicity	[70]
**110–114**	*Marinispora* sp. NPS12745	Sediment, the coast of San Diego, California	Antibacterial activity	[71]
**115–116**	*Saccharomonospora* sp. CNQ490	Marine sediment, the La Jolla Submarine Canyon	Cytotoxicity **116**: Antibacterial activity	[72,73]
**117–118**	*Nocardiopsis* CNS-653	Sediment sample, Fiji	TNF-R-induced NFκB	[74]
**119–120**	*Micromonospora* sp. CA-214671	Marine sediments, the Canary Islands	Antibacterial activity	[75]
**121–124**	*Actinomadura* sp. WMMB499	Ascidian *Ecteinascidia turbinata*	**123–124**: Nrf2-ARE activity	[76]
**125**	*Micromonospora* sp. WMMC-218	Ascidian *Symplegma brakenhielmi*, Florida, Stanblum State Park	Antibacterial activity	[77]
**126–127**	*Saccharomonospora* sp. KCTC-19160	Korean Collection for Type Cultures	BACE1 activity	[78]

## Data Availability

Not applicable.
